# A Brief Review of Carbon Dots–Silica Nanoparticles Synthesis and their Potential Use as Biosensing and Theragnostic Applications

**DOI:** 10.1186/s11671-022-03691-7

**Published:** 2022-06-04

**Authors:** Luis Fernando Ornelas-Hernández, Angeles Garduno-Robles, Abraham Zepeda-Moreno

**Affiliations:** 1 Onkogenetik/Mexicana de Investigación Y Biotectogía SA. de C.V., Av. Miguel Hidalgo y Costilla 1966, Guadalajara, Jalisco México; 2 Unidad de Biología Molecular, Investigación Y Diagnóstico SA de CV, Hospital San Javier, Pablo Casals 640, Guadalajara, Jalisco México; 3 Departamento de Clínicas Médicas, Centro Universitario de Ciencias de La Salud, Universidad de Guadalajara, Sierra Mojada 950, Guadalajara, Jalisco México

**Keywords:** Carbon dots, Nanoparticles, Carbon dots–silica nanoparticles

## Abstract

Carbon dots (CDs) are carbon nanoparticles with sizes below 10 nm and have attracted attention due to their relatively low toxicity, great biocompatibility, water solubility, facile synthesis, and exceptional photoluminescence properties. Accordingly, CDs have been widely exploited in different sensing and biomedical applications, for example, metal sensing, catalysis, biosensing, bioimaging, drug and gene delivery, and theragnostic applications. Similarly, the well-known properties of silica, such as facile surface functionalization, good biocompatibility, high surface area, and tunable pore volume, have allowed the loading of diverse inorganic and organic moieties and nanoparticles, creating complex hybrid nanostructures that exploit distinct properties (optical, magnetic, metallic, mesoporous, etc.) for sensing, biosensing, bioimaging, diagnosis, and gene and drug delivery. In this context, CDs have been successfully grafted into diverse silica nanostructures through various synthesis methods (e.g., solgel chemistry, inverse microemulsion, surfactant templating, and molecular imprinting technology (MIT)), imparting hybrid nanostructures with multimodal properties for distinct objectives. This review discusses the recently employed synthesis methods for CDs and silica nanoparticles and their typical applications. Then, we focus on combined synthesis techniques of CD–silica nanostructures and their promising biosensing operations. Finally, we overview the most recent potential applications of these materials as innovative smart hybrid nanocarriers and theragnostic agents for the nanomedical field.

**Graphical abstract:**

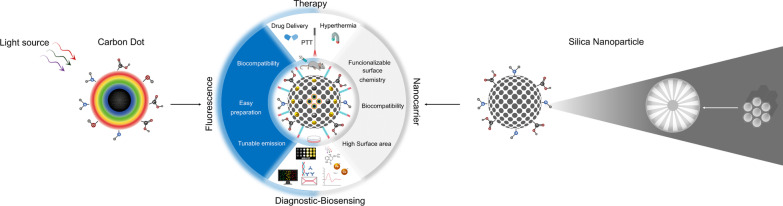

## Introduction

In recent years, the innovative field of nanotechnology has led to novel applications in diverse scientific disciplines due to the fascinating physical chemistry properties of nanosized materials (1–100 nm) and their interactions with other systems that exist at the molecular level (e.g., atoms and molecules) [1]. The development of devices at the nanoscale has revealed improved functions of multiple materials compared to their bulk version. Nanomaterials can be produced by using different physical (top-down) and chemical (bottom-up) synthesis methods, which can occur in the gas phase, liquid phase, and solid phase. Some examples of physical synthesis techniques include pulsed laser ablation in liquid (PLAL) [2], laser ablation, laser pyrolysis, microwave irradiation [3], chemical vapor deposition (CVD), physical vapor deposition (PVD), etching, electric explosion, high-energy ball milling, and electric arc deposition. Chemical synthesis techniques include the following: coprecipitation, hydrothermal synthesis, solvothermal synthesis, microemulsion, solgel, chemical reduction, spray pyrolysis, electrochemical synthesis, green synthesis, and biological synthesis. Selection of an appropriate nanomaterial synthesis method depends on various factors, such as available infrastructure, costs, performance, large scale, and ecofriendliness [4]. Additionally, each synthesis method allows obtaining specific nanomaterial characteristics and physical chemistry properties. These remarkable properties (surface area, optical, mechanical, magnetic, quantum effect, electrical conductivity, high thermal resistance) can be obtained by controlling the particle size and shape [5, 6]. Therefore, nanomaterials are excellent candidates to resolve, improve, or generate new promising applications in electronics, energy storage [7], biomedicine, biotechnology, pharmaceutical sensing, photocatalysis, and water remediation, among others [8, 9]. Regarding nanomedicine and biosensing, the most exploited nanomaterials properties are the optical, magnetic, and thermal properties and large surface area. For example, metallic nanoparticles known as plasmonic nanoparticles (containing gold, silver, copper, etc.) exhibit strong absorption in the visible region and a specific localized surface plasmon resonance (LSPR) [10]. This property of absorption and light scattering can be used for colorimetric sensing of biological species (e.g., DNA, aptamers, antibodies, proteins, carbohydrates, and lipids) [11] as well as for photothermal therapy (PTT), drug delivery and in vivo and in vitro imaging. In a similar manner, superparamagnetic nanoparticles (SMPs, e.g., Fe oxides and gadolinium) have been applied to the same applications mentioned above and as magnetic resonance imaging (MRI) contrast agents for the magnetic separation of biological moieties and induction of magnetic hyperthermia [12]. Other nanomaterials that present extraordinary properties for nanomedical and sensing applications are inorganic fluorescent nanoparticles [13]. A typical example of these nanomaterials is quantum dots (QDs), which are nanocrystals of semiconductor oxides and chalcogenides (e.g., ZnO, CdS, and CdSe). The fluorescence of QDs is related to the quantum confinement effect and the band gap, which produces a shift in the emission wavelength with respect to the particle size. In addition to QDs, other optical nanomaterials that have been developed for sensing and nanomedical applications include upconversion nanoparticles and, recently, CDs and graphene QDs. CDs are spherical nanostructures obtained by different synthetic methods using organic natural materials as synthetic precursors. The optical properties (high quantum yield, resistance to photobleaching, narrow emission, etc.) of all mentioned photoluminescent nanomaterials, including CDs, are notably superior to those of traditional organic dyes, which makes them suitable options for the design of highly sensitive biosensors. In contrast to QDs, CDs are obtained by straightforward methods that notably diminish the time and resources required for their production. Additionally, the outstanding optical properties of CDs significantly reduce the time required for obtaining reproducible results in sensing and optical assays, without the requirement of qualified personnel; moreover, these properties help to avoid the use of costly and complex equipment. On the other hand, owing to the biocompatibility, high surface area, tunable pore diameter, length, and spatial structure of silica-based nanomaterials, including nanoparticles and mesoporous structures, these materials are fundamental for the development of new multifunctional sensing and nanomedical devices.

The unique properties of the above-mentioned nanomaterials have motivated researchers to engineer complex nanostructures with different characteristics for multiple objectives. For instance, the creation of engineered hybrid nanostructures has been reported, where at least one of the elements is stimuli responsive, while the other has drug cargo properties. Such systems have shown improvements in drug delivery systems (DDSs) since the stimuli-responsive material allows drug release control both spatially and temporally. Some authors have engineered dynamic nanosystems that make use of “nanovalves” through self-assembly and supramolecular interactions. These tools act as open–close gates or “gatekeepers” that are activated by diverse stimuli (e.g., pH, redox potential, thermal, magnetic, and enzymatic stimuli) [14]. These outstanding mechanisms allow the production of nonburst, true controlled release systems.

CD–silica hybrid nanomaterials are ideal for the above-mentioned features and applications. First, CDs exhibit both outstanding optical properties and excellent biocompatibility. Moreover, silica nanomaterials are ideal nanocontainers for numerous species including small organic molecules, macromolecules, and nanoparticles. From this aspect, in this review, we will first discuss the synthesis and applications of CDs and silica nanoparticles. These two nanomaterials exhibit different properties. On the one hand, CDs have outstanding fluorescent properties; on the other hand, silica and its derivatives have the advantages of cargo capacity, large surface area, ordered pore structure, etc. Then, we summarize the combination of CDs/silica into one nanostructure (nanocomposite), the synthesis techniques and how some researchers have used this second-generation nanostructure for recent applications in biosensing and nanomedicine.

## Carbon Dots

Since the discovery of CDs through the electrophoretic purification of single-walled carbon nanotubes, which arose as by-products of the arc-discharge of soot [15], CDs have attracted attention due to their fascinating unusual physical and chemical features, such as emissive optical properties, high quantum yield, resistance to photobleaching, superior photostability, excellent water solubility, good biocompatibility, chemical inertness and low toxicity. Currently, it is known that the difference between CDs and their analogs, graphene QDs, is that CDs are quasispherical nanoparticles with diameters < 10 nm, composed of a ${\text{sp}}^{2}$ carbogenic core surrounded by an amorphous ${\text{sp}}^{3}$ carbon matrix, while graphene QDs possess higher crystallinity owing to the presence of mono- or multilayered graphite cores. Some experimental approaches have been proposed to elucidate the intrinsic photoluminescence generation mechanism of CDs, supported by theoretical computational modeling such as density functional theory (DFT) and time-dependent DFT calculations. Multiple authors suggest that unlike that of metallic QDs, the emission color of CDs does not depend on the nanoparticle size; instead, the emission is associated with different factors including the nature of the starting synthetic precursors, energy states, emissive traps, surface defects, and radiative recombination of excitons [16, 17]. Other authors claim that the emission is due to the formation of molecular fluorophores as side products during synthesis [18]; however, the nature of the photoluminescence is not yet clear [17, 19, 20]. Regarding chemical and biological aspects, the high content of carbon atoms and the heteroatoms in CDs confer them chemical characteristics such as high water solubility and, at the same time, grant them excellent biocompatibility.

### Carbon Dot Synthesis and Applications

The first characterizations of CD fluorescence showed that photoluminescence is an *excitation-independent emission* since CDs generally emit blue color, and depending on their superficial states, they might also emit another wavelength with lower intensity. Nevertheless, these emissions require different wavelength excitation sources. Therefore, to produce excitation-independent emission CDs that emit from a single wavelength excitation source, different strategies, such as engineering of surface chemistry and functional group heteroatom doping [21], have been applied. These approaches have been applied in conjunction with diverse top-down (e.g., microwave irradiation and laser ablation) and bottom-up (e.g., hydrothermal synthesis, carbonization, pyrolysis, and electrochemistry) synthesis methods [22–32]. Notably, CDs can be generated using organic natural sources and synthetic precursors that are selected to provide tunable fluorescence properties and functionalization for specific targeting ligands [33–38]. CDs have shown outstanding photophysical, photochemical, electroluminescent, and electrochemical properties. Some of these properties such as phosphorescence, photoluminescence, strong absorption, quantum confinement effects, high electrical conductivity, surface defects, and charge transfer effects have been exploited in diverse areas including biomedicine, forensic science, and electronic and storage and conversion of energy. In the last two areas mentioned, CDs have shown interesting promising applications in the fabrication of supercapacitors, rechargeable and lithium-ion batteries, light-emitting diodes (LEDs), organic light-emitting devices, luminophores, coreactants, solar cells, cathode and anodes, and in nanophotocatalysis, nano-organocatalysis, information encryption, and sensors [39, 40] (Table 1).

**Table 1:** Examples of common usable applications of CDs

Sensing applications	References
Be2 + ions detection	[21]
Selective detection of tartrazine in food samples	[34]
2,4,6-Trinitrophenol detection	[39]
Silver ions detection	[40]
*Energy and environmental applications*	
Photocatalysis degradation of naphthol blue black	[38]
Nanocatalysts oxidation of cyclohexane	[50]
Hypochlorite (ClO −) detection	[51]
Electroluminescent LEDs production	[59]
Photoluminescent LEDs production	[60]
Printing text and 3D printing	[41]
Solar cell devices	[17]

### Carbon Dots in Biomedical Applications

Due to the fascinating properties of CDs such as low cytotoxicity, high water solubility, biocompatibility and the photophysical and photochemistry properties mentioned in the previous section, these materials have been exploited in different biomedical applications. Examples include biosensors [41, 42], drug administration, allowing the controlled release of drugs [43], gene delivery, bioimaging, photoacoustic imaging, and photothermal and photodynamic therapy [44, 45] (Table 2) [46–53]. Kong et al. [54] produced functionalized CDs by using hydrothermal methods with phenylenediamine and ethylenediamine as precursors. Surface modification of CDs was used to promote electron transport. This investigation showed that the CDs were successfully employed as reactive oxygen species (ROS) scavengers in murine models, mimicking peroxidase (POD), catalase (CAT), and superoxide dismutase (SOD). These achievements demonstrated that CDs can be used in nanocatalytic medicine as excellent therapeutic agents for inflammatory liver diseases [50]. Yang et al. [55] developed CDs by a hydrothermal technique for use in gene delivery and cancer cell detection. The authors strategically incorporated polyethyleneimine (PEI) and folic acid (FA) into the CDs to facilitate biological interactions. The positively charged PEI molecules served as DNA-binding molecules, and folate served as a receptor. The nanomaterial demonstrated the ability to detect 293 T and HeLa cells and presented plasmid transfection activity [56, 57]. Recently, new trends in the applications of CDs have emerged. Naik et al. [58] produced pink fluorescent CDs for photomedicine. The authors showed that these pink fluorescent CDs can be applied for cancer cell bioimaging, antibacterial activity, and ROS scavenging. Another new topic regarding biomedical application of CDs is related to covering all spectral regions, particularly the near-infrared region [59–62]. Nevertheless, despite all these promising applications of CDs, to date, it is necessary to establish an effective large-scale production synthesis method, standardize the composition and structure of CDs, and achieve a better yield [63–69]. In addition, a better comprehension of the nature of the photoluminescence properties is needed [59, 70]. Li et al. [71] reported and interestingly achieved the large-scale production of CDs by an aldol condensation reaction at room temperature and ambient pressure. As a result, the authors produced a 1.083 kg CD batch.

**Table 2:** Biomedical applications

Starting precursor	Synthesis method	Use	Application	References
*Sargassum fluitans*	Hydrothermal	DNA detection	Biosensors	[72]
Urea/citric acid	Solvothermal	RNA detection		[73]
o-Phenylenediamine, 2-aminoterephthalic acid	Solvothermal	microRNA-21 detection		[56]
Citric acid, basic fuchsin	Hydrothermal	Intracellular pH sensing		[74]
Citric acid, ethylenediamine	Hydrothermal	Spectrofluorometric detection of cancer cells		[75]
Diammonium hydrogen citrate	Pyrolysis	*Escherichia coli* and iron(III) detection		[36]
Poly(ethylene glycol) (PEG800), cyanine dye	Solvothermal	Near-infrared fluorescence imaging and photothermal cancer therapy	In vivo and in vitro imaging	[76]
Citric acid, polyethyleneimine (PEI)	Hydrothermal	Lysosome labeling and imaging		[77]
Polyethyleneimine (PEI)	Hydrothermal	Gene/drug delivery/imaging		[78]
Citric acid, thiourea, 3-aminophenylboronic acid	Hydrothermal	Glucosamine and liver cancer cell imaging		[79]
Carrot	Hydrothermal	Ratiometric two-photon fluorescence turn-on sensing of sulfide anion in biological fluids		[37]
Polyethyleneimine (PEI), folic acid (FA)	Hydrothermal	Gene therapy	Drug and gene delivery	[55]

## Silica Nanoparticle Synthesis and Applications

The obtention of silica nanoparticles involves the well-known solgel processing method, in which hydrolysis and condensation reactions (see Eq. 1 and Eq. 2) of common metal alkoxides (e.g., tetraethyl orthosilicate (TEOS) and tetramethyl orthosilicate (TMOS)) take place, either under acidic or under basic conditions. Additionally, other precursors such as transition metals (Ti and Zr), alkoxides, aluminates, and borates can be used. By controlling a series of factors, such as reaction time, gelation, aging, and drying, this synthetic route can produce colloidal silica, oxide films, gels (aerogels and xerogels), or ceramic materials [80].

Hydrolysis:

1 $${\text{Si}}({\text{OR}})_{4} + {\text{H}}_{2} {\text{O }} \to {\text{ HO}} - {\text{Si}}({\text{OR}})_{3 } + {\text{ ROH}}$$

Condensation:

2 $${\text{(OR}})_{3 } {\text{Si}} - {\text{OH}} + {\text{ HO}} - {\text{Si}}({\text{OR}})_{3} { } \to { }({\text{OR}})_{3 } {\text{Si}} - {\text{O}} - {\text{Si}}({\text{OR}})_{3 } { } + { } {\text{H}}_{2} {\text{O }}$$

One of the most important methods for silica nanoparticle production was reported by Stöber et al. [81]. The authors were able to produce monodispersed silica spheres with diameters of a few microns through the hydrolysis of alkyl silicates in a mixed solution of alcohol and ammonia. Over the years, this synthesis method has been modified or adapted for different purposes [82]. For example, compared to the traditional Stöber method, the use of the Stöber method in combination with an inverse microemulsion system produces smaller and more monodisperse silica nanoparticles. This mixed synthetic route will be discussed later. Some properties of silica nanoparticle include [82–85] easy surface functionalization, [86–89] chemical inertness, low toxicity, light transparency, and good water solubility. These properties have promoted silica-based nanomaterials as potential candidates for engineering hybrid multifunctional nanostructures for biomedical applications [90]. For instance, numerous researchers have created diverse functionalized and doped silica nanostructures through the Stöber et al. [81] method, inverse microemulsion [91–93], and one-pot synthesis, generally in combination with semiconductor QDs, metallic nanoparticles, organic fluorescent dyes, functional molecules, and drugs as doping agents [94–97]; these approaches have resulted in multifunctional fused nanostructures with multiple morphologies such as core–shell, multicore–shell, sesame ball, hollow and yolk–shell structures [98], which have exhibited great effectiveness for drug delivery, plasmid cell transfection, and theragnostic applications (Table 3) [99–101].

**Table 3:** Diverse synthesis routes for silica nanoparticles and their applications

Synthesis method	Biosensing/theragnostic	References
Solgel	Chemically reactive surface and FITC encapsulation for potential bioimaging device	[102]
Modified Stöber	Fluorescent silica nanoparticle for high-resolution STED and confocal microscopy	[103]
Modified Stöber	Fluorescent silica nanoparticles for cervical cancer cell line imaging	[85]
Inverse microemulsion	Gene delivery	[104]
Modified Stöber	Chemical sensing	[105]
Ultrasonication solgel	Drug delivery and gene delivery	[106]
Modified Stöber	Targeted cell imaging	[107]
Inverse microemulsion	Leukocyte counting with a sheath-flow-free microchip flow cytometer	[108]

### Mesoporous Silica Nanoparticle Synthesis and Applications

In recent decades, many clever efforts have been made to improve and exploit the unique physicochemical properties of silica, which has been reflected in an exponential increase in the number of distinct applications. In this context, some mesoporous silica nanomaterials have emerged from the combination of various synthesis methods, particularly combining solgel chemistry with templating methods by using cationic, anionic surfactants and triblock copolymers as templates [109–111]; these methods generally yield diverse zero-dimensional, one-dimensional, two-dimensional, and three-dimensional (3D) mesoporous nanostructures that exhibit unique physicochemical properties such as high thermal stability, resistance to corrosion, large surface area, ordered pore structure, easy surface functionalization, and tunable pore volume and particle size. It is worth mentioning the pioneering work by Mobil researchers [112], who produced ordered hexagonally mesoporous nanostructures called MCM-41 through a surfactant-templated procedure assisted by hydrothermal treatment and further calcination. Similarly, Zhao [113] created well-ordered hexagonal mesoporous silica structures called SBA-15 through triblock copolymer synthesis (PEO-PPO-PEO) using amphiphilic block copolymers and the cationic surfactant cetyltrimethylammonium (CTA +) under acidic conditions as organic structure-directing agents (organic templates). Depending on the synthetic method used, complementary steps are required to develop mesoporous nanomaterials, for instance, by removing the surfactant template through calcination, acidic washing, or solvent extraction. Inherently, the governed self-assembly interaction and the reaction conditions (e.g., surfactant concentration, silica precursor, pH, and temperature) can control the morphology, surface area, and pore size and volume.

Mesoporous silica materials can be synthesized with diverse morphologies and mesostructures. The very well-known M41S family, for example, consists of diverse geometric structures (e.g., MCM-41, MCM-48, and MCM-50) that, in conjunction with SBA-15, are typical mesoporous silica materials, which have 2-hexagonal honeycomb-like porous structures, 3D-cubic structures, and lamellar structures [114]. Regarding the morphology, mesoporous nanostructures with different geometries have been produced, including hollow spheres, rods, gyroids, fibers, and helical fibers, which have been associated with the fine control of the reaction system [98]. A variety of synthetic routes for mesoporous silica nanoparticles have been proposed. The most common synthesis techniques are based on the modified Stöber method and on the soft template method, which produce hollow silica materials employing various soft templates such as amphiphilic surfactants, micelles, microemulsion droplets, and vesicular structures. On the other hand, the hard template method has also been used. This method is based on the use of polymer beads such as polystyrene (PS) particles and metal or oxide nanoparticles. Finally, an aerosol-assisted synthesis method has also been described [115].

The unique characteristics of mesoporous silica (i.e., large surface area, pore diameters usually from 2 to 50 nm, and surface functionalizability) are the driving force for interesting catalysis, adsorption, and sensing applications. These important properties have allowed the synthesis of silica hybrid nanostructures for drug delivery with increased loading and release rates, combining fluorescent and therapy molecules, and these hybrid systems have been used for catalysis and sensing recognition (Table 4) [116–118]. The use of hybrid mesoporous silica nanomaterials has improved current DDSs based on pure silica materials [119–122]. The motivation behind the development of these hybrid nanosystems is founded on the fact that the pore tunnels of mesoporous silica can be loaded with anticancer drug molecules and fluorescent contrast agents and then arranged dynamically by sealing the pores through stimuli-responsive supramolecular interactions employing matching molecules, or even nanoparticles, which can act as “gatekeepers.” The on-demand open–close response of such gatekeepers can be achieved by using either exogenous stimuli such as light, temperature, electricity, or magnetic field or endogenous stimuli such as pH gradients, enzymes, and temperature changes. In this way, systems with true control over the drug release kinetics are obtained [115, 123–129].

**Table 4:** Diverse synthesis routes for mesoporous silica nanoparticles and applications

Synthesis method	Biosensing/theragnostic	References
Modified Stöber	MRI contrast agent and optical sensing for in vivo and in vitro applications	[130]
Modified Stöber	Tumoral LNCaP cells selectivity and internalization	[131]
Modified Stöber	Nanotheranostics for camptothecin delivery and multimodal imaging	[132]
Modified Stöber	Drug delivery and hyperthermia	[133]
Modified Stöber	Removal of sodium dodecylbenzenesulfonate	[134]
Modified microemulsion assisted by the diffusion of metal precursors	Potential application for catalysis, optics, or nanomedicine	[135]
Single-micelle templating	Antimicrobial applications	[136]

Recently, some authors have gone beyond the limits of common morphologies of mesoporous silica nanoparticles (e.g., hollow spheres, fibers, rods, and spherical nanoparticles) due to their inherent limitations in surface area accessibility, which significantly diminishes the loading capacity of molecules of interest. Typically, mesoporous silica nanostructures are limited to small molecule cargo due to the uncontrolled synthesis of materials with large pore sizes [137]. Thus, to overcome this limitation, engineered dendritic silica particles have recently been fabricated. For instance, Polshettiwar et al. [138] synthesized fibrous silica nanospheres (KCC-1) using cetylpyridinium bromide (CPB) or cetyltrimethylammonium bromide (CTAB) as templates and a microwave-assisted hydrothermal technique. The nanospheres showed a high surface area produced by the dendritic silica fibers as well as advantageous thermal, hydrothermal, and high mechanical stabilities that can be used for extended applications [111, 139, 140]. On the other hand, 3D-dendritic mesoporous silica nanospheres that contain a center-radial mesostructure have also been produced by chemical methods. The synthesis was achieved by using the biphase stratification method. This synthetic route can control the large pore size and thickness of mesoporous nanospheres, which were successfully confirmed to be protein-releasing nanocarriers. In addition, this synthesis method allowed the construction of uniform mesoporous core–shell nanostructures with Au nanoparticles and Ag nanocubes as cores with 3D dendritic mesoporous radial channels [111, 141, 142]. These silica nanostructures exhibited a high surface area and an increased protein loading capacity due to control of the structural characteristics through tuning of the particle size, pore size length, and thickness. The physicochemical properties of these materials suggest their feasibility for multiple useful applications [143–146].

## Carbon Dot–Silica Nanoparticle Synthesis and Applications

One of the first approaches regarding the combination of carbon/silica gel was carried out in 1998 by Nobosuke et al. [147] [148]. By using polycyclic aromatic compounds as graphite precursors, the solgel method and calcination, the authors produced a carbon/silica gel nanocomposite that showed interesting photoluminescence properties. They suggested that the carbon contained within the nanocomposite was the key factor regarding the green luminescence, while the blue luminescence was generated by the silica gel matrix. The authors concluded that the pi-electron conjugated system and the photoluminescence required further study. To the best of our knowledge, Nobosuke’s study was pioneering in the production of fluorescent carbon/silica nanocomposites. Currently, there are variations in the fabrication methods of carbon/silica nanocomposites, principally because the source of fluorescent carbogenic elements is now provided by CDs, which can be presynthesized separately. Moreover, new variations have been achieved through distinct synthesis techniques, where the CDs have been grafted or doped in a silica matrix, forming a single nanostructure. In the following sections, we review some of the most interesting methods proposed for the synthesis of CD–silica hybrid nanostructures.

Recently, most of the strategies focusing on producing nanocomposites or nanoparticles of carbon/silica have used CDs and silica precursors [149, 150]. The most commonly used synthesis method involves the combination of various techniques, for example, the typical synthesis of mesoporous silica plus the addition of presynthesized CDs. In some cases, CDs can be synthesized in situ with mesoporous silica [151–153]. For the creation of multifunctional CD–silica nanoplatforms, it is highly important to design a suitable functionalization and bioconjugation strategy to obtain successful interactions and good results in theragnostic, biomedical, and sensing applications [53, 57, 67], given that mixing two distinct nanoparticles and linking with other different components (reactive groups, organic and inorganic components, and biomolecules) requires good knowledge of conjugation chemistry, click chemistry, and supramolecular chemistry. Engineering a suitable linking strategy depends on the intended objective and the order of the involved components. In this case, silica is the best candidate for functionalization due to its facile surface chemistry and capacity to assemble a few layers through the linkage of silane coupling agents. Many silane coupling agents exist, and some of the agents most commonly used in theragnostics and biosensing include (3-aminopropyl)trimethoxysilane (APTMS), (3-aminopropyl)trimethoxysilane (APTES), 3-mercaptopropyltrimethoxysilane (MPTMS), 3-(trihydroxysilyl)propyl methylphosphonate monosodium salt solution (THMPS), and TMOS. Some of these siloxane precursors not only grant functional groups to facilitate functionalization or bioconjugation but also, in some cases, can serve as colloidal stabilizers [154–158].

CD–silica nanostructures have attracted the interest of the scientific community, especially materials scientists, since combining CDs with silica in a single hybrid functional nanostructure results in a novel smart approach to exploit the physicochemical properties of both materials concomitantly for different potential applications (Tables 5 and 6) [159–165].

**Table 5:** Diverse synthesis routes for silica–CD nanoparticles and biosensing applications

CD synthesis method	Synthesis method for silica–carbon nanoparticles	Biosensing/theragnostic	Refs.
Organosilane pyrolysis	Organosilane pyrolysis	Vanadium(V) detection	[166]
One-pot solvothermal	One-pot solvothermal	Fe3 + detection and cancer/normal cell differentiation	[167]
Organosilane pyrolysis	Stöber/molecularly imprinted silica	Sensing of rhodamine 6G	[168]
Microwave irradiation	Reverse microemulsion	Photocatalyst for highly selective solar fuel production from CO_2_	[169]
One-pot copolycondensation/solgel	One-pot copolycondensation/solgel	Temperature sensing and cell labeling	[170]
Hydrothermal	Solgel	Cu2 + ion detection	[171]
Organosilane pyrolysis	Solgel	Nitride sensing in food	[172]
One-step hydrothermal method	Solgel/molecularly imprinted silica	Anti-inflammatory drug celecoxib detection	[173]

**Table 6:** Diverse synthesis routes for hybrid carbon dot/silica/magnetic nanoparticles and bioapplications

CD synthesis method	Synthesis method for magnetic nanoparticles	Synthesis method for silica–magnetic nanoparticles	Biosensing/theragnostic	References
Organosilane pyrolysis	High-pressure hydrothermal	Solgel/surfactant template	Potential bioimaging sensor	[253]
Hydrothermal	Solvothermal method	Solgel/surfactant template	Drug delivery, fluorescence, and MRI	[230]
Hydrothermal	Coprecipitation method	Solgel	Fluorescence sensing, magnetic separation, and live cell imaging of fluoride ions	[254]
Hydrothermal	Solvothermal method	Reverse microemulsion/molecularly imprinted polymer	2,4,6-Trinitrophenol (TNP) detection	[255]
Calcination	Calcination	Solgel/surfactant template and calcination	Fluorescence and magnetic contrast agent for in vivo and in vitro applications	[130]

Diverse synthesis routes for hybrid carbon dot/silica/gold nanorod nanoparticles and bioapplications				
CD synthesis method	Synthesis method for gold nanorods	Synthesis method for silica-gold nanorods	Biosensing/theragnostic	Reference
One-step hydrothermal method	Seed-mediated growth method	Solgel	Atherosclerosis detection	[256]

### Molecular Imprinting Technique

**Fig. 1:**
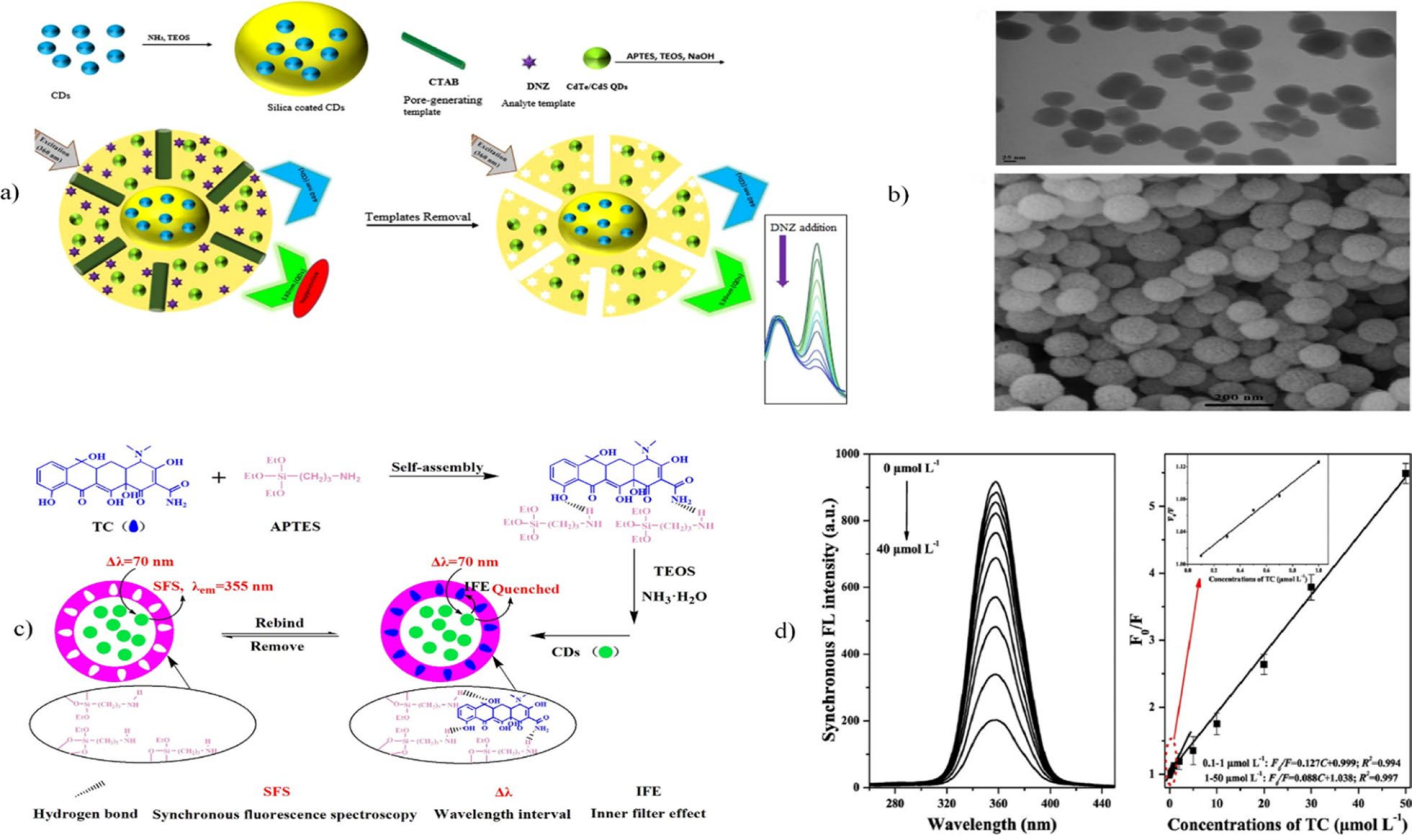
Representative schemes for the construction and function of CD–silica nanosensors: **a** Scheme of nanosensor construction and operation for diniconazole detection, **b** SEM and TEM micrographs of the mMIP@CDsQDs. Adapted with permission from ref. [180] copyright 2017 Elsevier. **c** Scheme of nanosensor construction and operation for tetracycline detection, **d** SFS spectra showing the fluorescence intensity behavior as a function of the TC concentration. Adapted with permission from ref. [188] copyright 2017 Elsevier

The molecular imprinting technique (MIT), which produces molecularly imprinted polymers (MIPs), is a synthetic process that involves host–guest supramolecular systems. This technique is based on the bonding of a polymer (host) to a target molecule (guest). The target molecule interacts either covalently or noncovalently with specific functional groups from the host–polymer matrix; subsequently, the target molecule is removed from the polymer, creating an interacting cavity or template, which is then able to rebind a new guest target molecule when the MIP is mixed with a sample that contains the target molecule [174, 175]. There are three typical synthesis routes to obtain MIPs: (i) synthesis from monomers in the presence of a template, (ii) phase inversion; and (iii) soft lithography. MIPs can be applied for biomarker sensing, pathogen detection, and nonsensing applications such as chiral molecule detection, catalysis, and specific separation processes [176, 177]. Recently, numerous approaches have been developed to fabricate multipurpose nanoplatforms by using the MIT combined with fluorescent signal molecules or targeting molecules. Additionally, engineered MIPs have been used as molecular recognition sensors of different analytes such as biomolecules, ions, and chemical and biological moieties [178, 179]. For example, Amjadi and Jalili [180] prepared a multiemission mesoporous multilayered structure constituted by semiconductor QDs, CDs, and silica shells. The employed route to obtain the materials was performed through a one-pot method assisted by the Stöber solgel synthesis and MIT. The preparation of the nanoprobe was carried out as follows: Previously synthesized carbon QDs were covered with a layer of silica; later, by cocondensation, a layer of mesoporous silica was formed on the layer that covered the carbon QDs. In this step, diniconazole (DNZ), organic QDs, and CTAB were added, with the last reagent added to make the layer porous. Finally, the DNZ and CTAB templates were removed by washing with water and methanol (see Fig. 1a). The authors obtained spherical nanoparticles that showed a rough surface, as observed by scanning electron microscopy (SEM). Similarly, transmission electron microscopy (TEM) analysis revealed nanoparticles with diameters of 100 ± 10 nm (see Fig. 1b)**.** Due to the multiple emission at 470 nm and 530 nm conferred by both QDs, the nanoparticles were used as a ratiometric sensor for the recognition and detection of DNZ, analog molecules and real samples of DNZ in soil, water, and wastewater. To test the sensor behavior, the probe performance was evaluated at different pH values, observing that within the pH range of 6.0–9.0 without analyte, the intensity ratio was not altered, while in the presence of DNZ and at pH 8.0, the intensity ratio was changed. Therefore, the fluorescence intensity showed high sensitivity to DNZ concentration, with a good linear relationship. To understand the relation of fluorescence quenching as a function of DNZ quantity, the binding kinetics of the template were tested versus time. The authors suggested that the higher adsorption of DNZ on the nanoprobe was due to the greater number of recognition sites generated on the pores of the mesoporous shell. These recognition sites were able to rebind the DNZ molecules. Therefore, the authors described that the fluorescence quenching behavior was related to the influence of the functional amine groups that act as acceptors for photogenerated holes in QDs [181, 182]. To verify the efficiency of the sensor, it was compared to common detection techniques, exhibiting a detection limit equivalent to that of ELISA and chromatographic assays, high reproducibility, and stability.

Concerning environmental issues, the combination of mesoporous silica and CDs as hybrid nanomaterials has been employed as a versatile alternative to detect diverse ambient molecules and compounds harmful to human health [153, 178, 183–186]. Clearly, the high quantum yield, low cytotoxicity, tunable fluorescence, high surface area, tunable pore size, and mesoporosity are the outstanding properties exploited to construct these nanosensors. For instance, Liu et al. [187] addressed the detection of bisphenol A (BPA), an endocrine-disrupting organic molecule found in food packaging. In particular, the nanomaterial was obtained by using a MIT and in situ hydrothermal synthesis; the CDs were synthesized through a pyrolysis decomposition method using anhydrous citric acid and AEAPMS as the precursor source. Since MIP-coated CDs were fabricated by solgel synthesis in the presence of a BPA template, TEOS and APTES were used to generate BPA binding sites for recognition. The templates were then removed by the solvent elution method with water/ethanol. In a simple manner, the authors created a highly specific BPA nanosensor, whose sensing principle was based on the fluorescence quenching of CDs, with a reliable concentration sensitivity of 100 nM to 4200 nM and nonspecific detection of interfering ions and phenolic compounds. The nanosensor results were comparable with high-performance liquid chromatography (HPLC) detection and showed a similar extrapolated performance when real river water samples were analyzed.

Another similar study was reported by Yang et al. [188]. First, through hydrothermal synthesis, they obtained nitrogen-doped CDs by using citric acid and urea as precursors, and then, a modified inverse microemulsion system (cyclohexane/n-hexanol/Triton X-100/water/CDs) was used to encapsulate the CDs by polymerization of TEOS and APTES. Finally, the analyte was added as a template and removed by washing to create the nanostructure MIP@CDs (see Fig. 1c). The regular spherical nanoparticles obtained were applied as sensors to detect the antibiotic tetracycline (TC). Based on synchronous fluorescence spectroscopy (SFS), the fluorescence quenching induced by the compound was investigated. The results suggested that TC is physically adsorbed on the MIP@CDs, producing a quenching effect of the synchronous fluorescence spectra dependent on the concentration of TC. As complementary deductions, due to the minimal change in fluorescence lifetime observed, the quenching mechanism is related to the inner filter effect (IFE) and not Förster resonance energy transfer (FRET) (Fig. 1d).

**Fig. 2:**
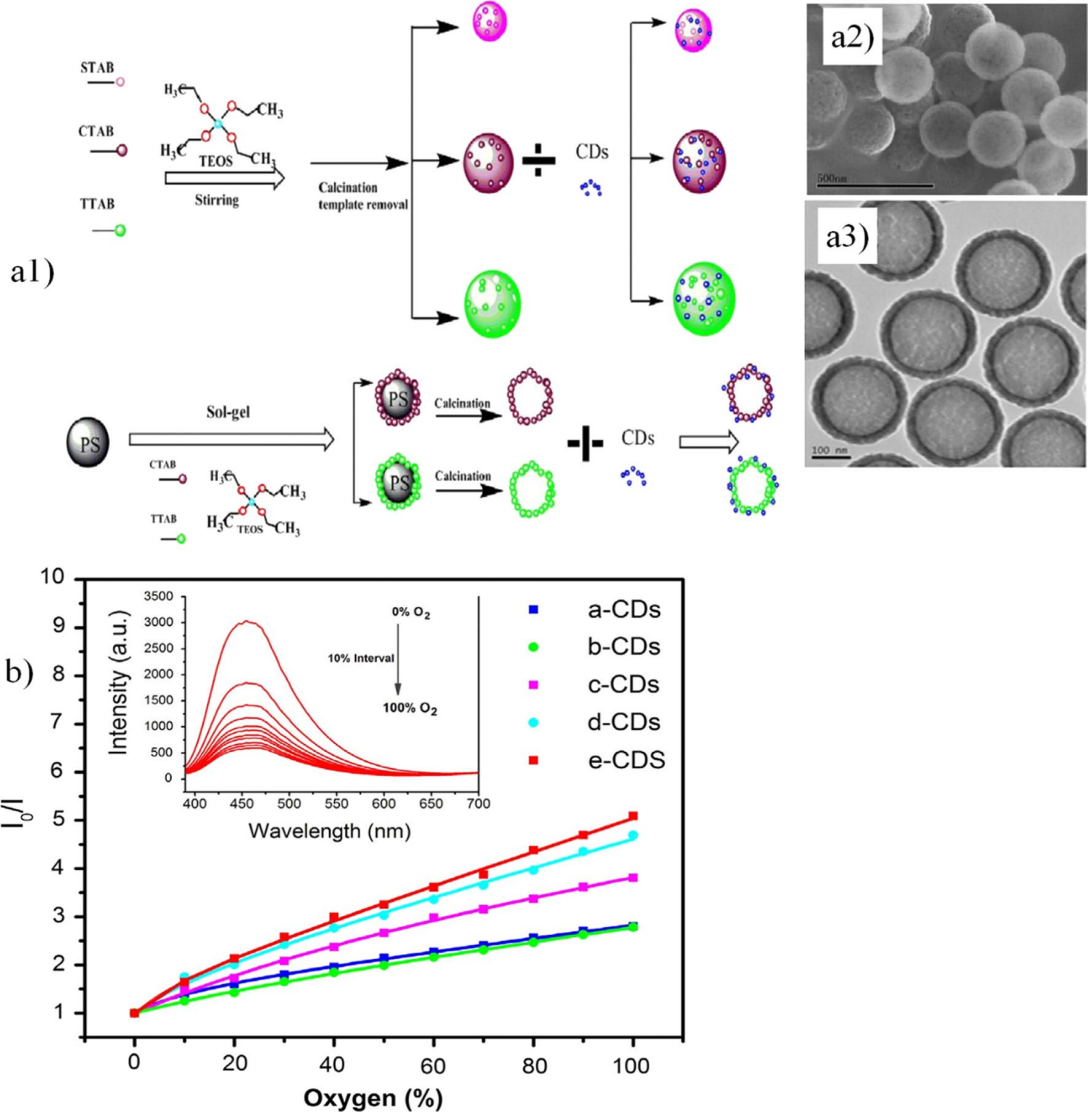
**a1** Scheme of the construction of the nanosensor for oxygen sensing, **a2**, **a3** FE-SEM and TEM micrographs showing the morphology of the nanoparticles, **b** graph presenting the variation in fluorescence intensity as a function of the concentration of oxygen, and Stern–Volmer plot showing the quenching degree of the luminophore. Adapted with permission from ref. [190] Copyright 2016 Elsevier

Due to their high surface area, pore volume, surface functionalization, and relatively thick wall, mesoporous nanomaterials have been used for gas sensing [189]. Ordered mesoporous materials allow the effective hosting of diverse molecules. For example, Wang et al. [190] obtained a feasible oxygen sensor by grafting CDs in hollow mesoporous silica microspheres (HMSMs), as well as in mesoporous silica microspheres (MSMs). Briefly, anhydrous citric acid and the silane coupler KH-602 were used as precursors for the formation of CDs through the pyrolysis method [190]. On the other hand, MSMs were synthesized by using the surfactants CTAB, tetradecyltrimethylammonium bromide (TTAB), stearyltrimethylammonium bromide (STAB), and triethanolamine (TEA) as cotemplates and TEOS as a polymerizing agent. The process was completed by autoclaving at 383 K and subsequent calcination at 823 K to remove the templates. To fabricate HMSMs, first, PS microspheres were fabricated using polyvinylpyrrolidone (PVP), $\alpha ,\alpha^{,}$-azodiisobutyramidine dihydrochloride (AIBA), and styrene through heating in a water bath. PS microspheres were added to a mixture of TEOS, CTAB, TTAB, ethanol, and ammonium hydroxide, which acted as a basic catalyst. Then, the mixture was heated and calcined at 823 K. Finally, the produced HMSMs were loaded with CDs for further testing (see Fig. 2a1). The size and shell thickness of the obtained HMSMs were dependent on the surfactant concentration and ratio; moreover, the surfactants CTAB and TTAB, used as structure-directing agents, tended to increase the pore number and particle size (see Fig. 2a2 and a3). The dependence of the fluorescence intensity of loaded CDs-MSMs and CDs-HMSM as a function of analyte concentration was exploited for oxygen sensing; the results suggested that the emission intensity is oxygen concentration dependent, showing a decrease in intensity with higher oxygen concentrations because of a quenching effect. Significantly, the probe showed a response and recovery emission intensity behavior, validated by the nitrogen–oxygen replacement mechanism; additionally, the hybrid nanostructures exhibited a short response time and recovery time of 4.85 and 23.23 s, respectively, which are desirable for oxygen sensors (see Fig. 2b**)**.

### Carbon Dot–Silica Nanoparticles by Inverse Microemulsion

In 1981, the term “microemulsion” was defined as a system of water, oil, and an amphiphile that altogether is a single optically isotropic and thermodynamically stable liquid solution [191]. In recent years, diverse descriptions of microemulsions have been reported by chemists, physicochemists, and engineers. The microemulsion formulation is related to the intrinsic physicochemical characteristics that dictate the multifactorial requirements to form a stable microemulsion system [192], which is typically composed of a ternary system (water/oil/surfactant) or quaternary system (water/oil/cosurfactant (usually short-chain alcohols)/surfactant) [193]. Regularly, microemulsions form spontaneously, and they exhibit thermodynamic stability because the free energy of the microemulsion is lower than that of the separated states [194]. In a microemulsion system, primarily, the surfactant molecule is adsorbed at the oil–water interface, reducing the interfacial tension and conferring stability to the dispersed phase. In microemulsions, the self-assembly of surfactant molecules produces discrete surfactant aggregates that are topologically diverse (i.e., spherical micelles, cylindrical micelles or rodlike, vesicular, lamellar, and reverse micelles) [195, 196]. The shape and size of these supramolecular structures depend on multiple factors such as the hydrophilic–lipophilic balance (HLB), critical micellar concentration (CMC), and surfactant packing parameter ratio (${\text{N}}_{{\text{s}}} = {\text{v}}/{\text{a}}l_{c}$) [197–199]. These aggregates are in the nanosize range and can be exploited as nanoreactors. Specifically, reverse micelles or inverse microemulsions have been used as nanoreactors or “water pools” for the synthesis of various nanomaterials such as QDs and metallic and polymeric nanoparticles [200–205]. Usually, two reactants are placed in separated microemulsion systems; then, both microemulsions are combined, causing droplet collision and fusion and, later, fission, intermixing the reactants. Afterward, the reaction occurs inside the nanoreactor or water pool [206]. The radius of the water pool can be controlled by varying the water/surfactant ratio, ${\text{W}}_{0}$, which allows the synthesis of monodisperse nanoparticles and represents a suitable option to control the particle size because the compartmentalized nanodroplets limit the nucleation, growth, and coalescence aggregation of as-synthesized nanostructures [207]. Silica nanoparticles can be produced with inverse microemulsions, with smaller sizes (below 100 nm) than those obtained by the Stöber method [208–214].

**Fig. 3:**
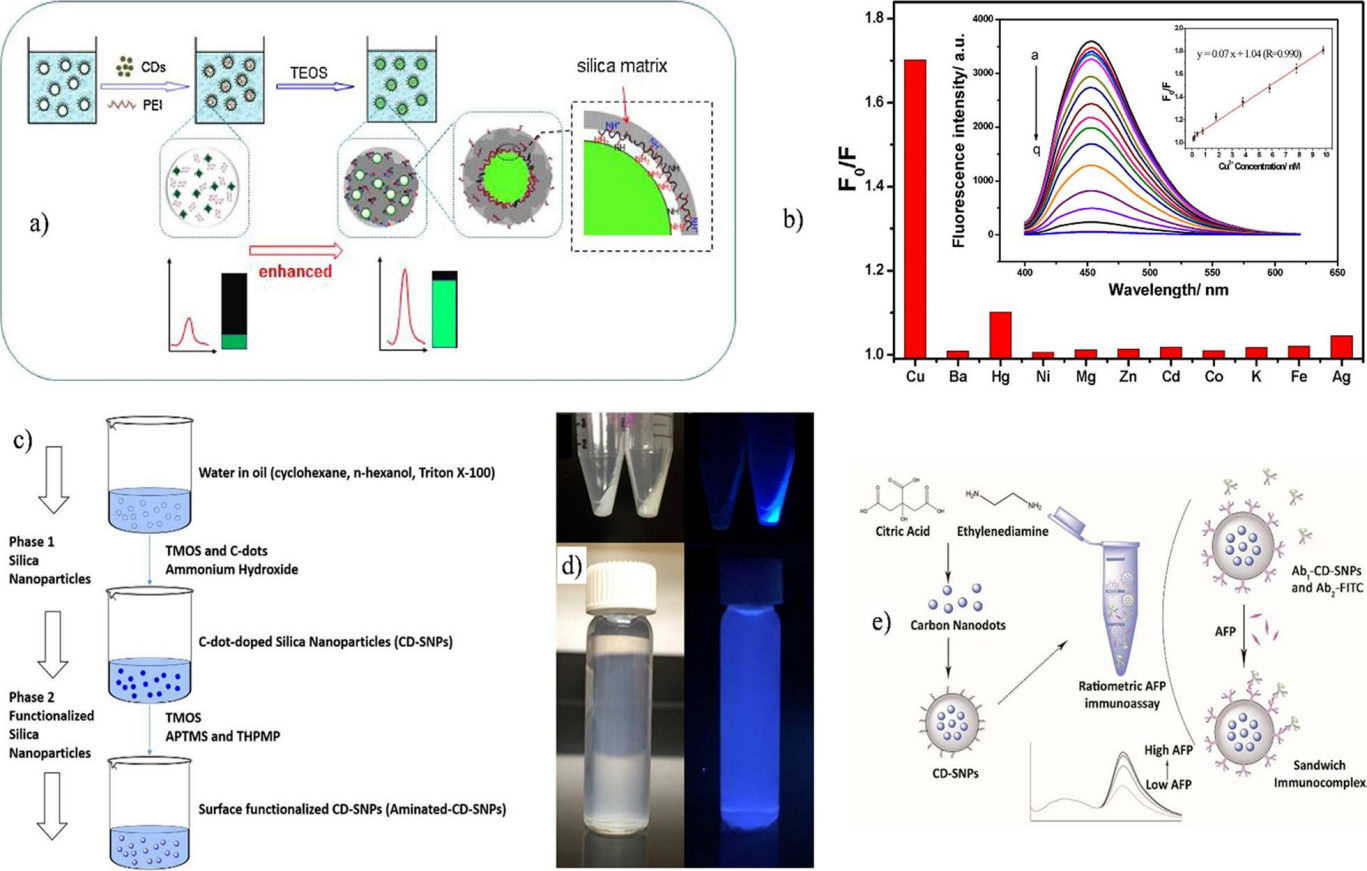
Schematic view of the fluorescence enhancement mechanism of carbon dots (**a**) and graphs showing the fluorescence intensity decrease in the presence of copper ions and selectivity over other metal ions (**b**). Adapted with permission from ref. [219] © 2017 Elsevier. Schematic illustration showing the microemulsion system used to obtain CDs/silica (**c**) and CDs/silica under UV light (**d**). CDs/silica as a ratiometric immunosensor for alpha-fetoprotein detection (**e**). Reproduced with permission from reference [223] © 2016 Royal Society of Chemistry

The robust intrinsic properties of CDs, such as high photochemical stability, low toxicity, high quantum yield, and electron acceptor or donor ability, make them potential candidates to detect metallic analytes [166, 215–218]. For instance, Qiao et al. [219] exploited these properties, developing enhanced fluorescence CD–silica nanoparticles to detect cupric ions (${\text{Cu}}^{2 + }$). The authors produced doped silica nanoparticles with CDs through a one-pot synthesis. First, the passivated CDs were obtained by a hydrothermal pyrolysis technique by using citric acid and PEI as precursors, while to produce doped silica nanoparticles, a solution of PEI-passivated CDs was added to a tailored inverse microemulsion system of TX-100/cyclohexane/1-hexanol/ultrapure water, followed by the addition of TEOS and ammonium hydroxide to the system. The investigation showed interesting results about CD quantum yield improvement. The addition of PEI to the system was intended to graft or cargo the CDs into the silica matrix through supramolecular interactions between PEI amino groups and CD carboxyl groups. The results demonstrated that an optimized PEI concentration not only helped to incorporate the CDs into the silica but also improved the fluorescence quantum yield from 13.7% in free-state CDs to 38.6%. The authors suggested that the possibly improved fluorescence was attributed to the interaction of positively charged tertiary amine groups present on PEI molecules with negatively charged ${\text{SiO}}^{ - }$ groups, while the primary and secondary amines were concentrated and directed to the surface CDs facilitating the supramolecular interaction between carboxyl groups and amine groups (see Fig. 3a). As the authors noted, fluorescence enhancement is due to the aforementioned supramolecular interactions; thus, they used this behavior to create a fluorescence turn-off system by disrupting these interactions with metallic analytes. The results showed that the doped silica nanoparticles have higher selectivity for detecting copper ions since they can form complexes with the amine groups, thereby destroying supramolecular interactions and resulting in a decrease in fluorescence intensity (see Fig. 3b).

Regarding CDs used for biorecognition, various strategies have been exploited [77, 167]. The fluorescence versatility combined with the well-documented silica chemistry allows the creation of nanomaterials for specific biodetection purposes [220–222]. Wu et al. [223] prepared CD-doped functionalized silica nanoparticles to detect alpha-fetoprotein, a glycoprotein related to liver diseases. CDs were obtained through the hydrothermal synthesis of citric acid and ethylenediamine. Then, the fluorescent dots were encapsulated in a silica shell by using an inverse microemulsion system, with TMOS, 3-(trihydroxysilyl)propyl methylphosphonate (THPMP), and APTMS used as polymerizing agents (see Fig. 3c and d). THPMP was added to provide negatively charged phosphate groups, avoiding colloidal instability or agglomeration, while APTMS was used to graft positively charged amino groups as active binding sites. Subsequently, these nanoparticles were covalently bonded to anti-alpha-fetoprotein through glutaraldehyde cross-linking to build an ${\text{Ab}}_{1} - {\text{CD}} - {\text{SNPs}}$ component, which was later mixed with a second component of secondary anti-alpha-fetoprotein ${\text{Ab}}_{2} - {\text{FITC}}$, forming an immunocomplex. The immunosensor showed dual emission at 440 and 550 nm, with the first peak due to the CDs and the second from FITC. This multiemission system was successfully used to detect alpha-fetoprotein, obtaining a good linear relationship of 0.317–280 µg/dl with a correlation of ${\text{R}}^{2} = 0.997$ (see Fig. 3e).

Concerning the nanomedicine field, several researchers have created distinct nanomaterials for applications in biological systems. Fluorescent, magnetic, polymeric, and metallic nanoparticles have been used for bioimaging, drug delivery, and therapy. In this sense, the nanoparticles can be combined with organic and inorganic moieties to form theragnostic nanoplatforms [151, 224–229]. Some of these nanoplatforms have been a hot research topic in nanomedicine because of their improvement in diagnosis and therapy in biological system applications (Table 6).

For instance, Das et al. [230] developed a theragnostic nanoplatform that exploits the intrinsic properties of nanomaterials, such as superparamagnetism, high fluorescence, mesoporosity, and high surface area [231]. Based on supramolecular interactions, they developed a host–guest system capable of assembling different nanomaterials. The prepared nanoplatforms were obtained through diverse synthesis and assembly steps. First, magnetic gadolinium and iron oxide nanoparticles were synthesized by a solvothermal method. Then, through the cationic surfactant CTAB and the polymerizing agents TEOS and APTS, the magnetic nanoparticles were coated with silica layers, creating a core–shell structure. Later, the nanoparticles were functionalized with positively charged amine groups, which served to bind negatively charged boronic acid-functionalized CDs (BNSCQDs) that had been previously synthesized [79].

**Fig. 4:**
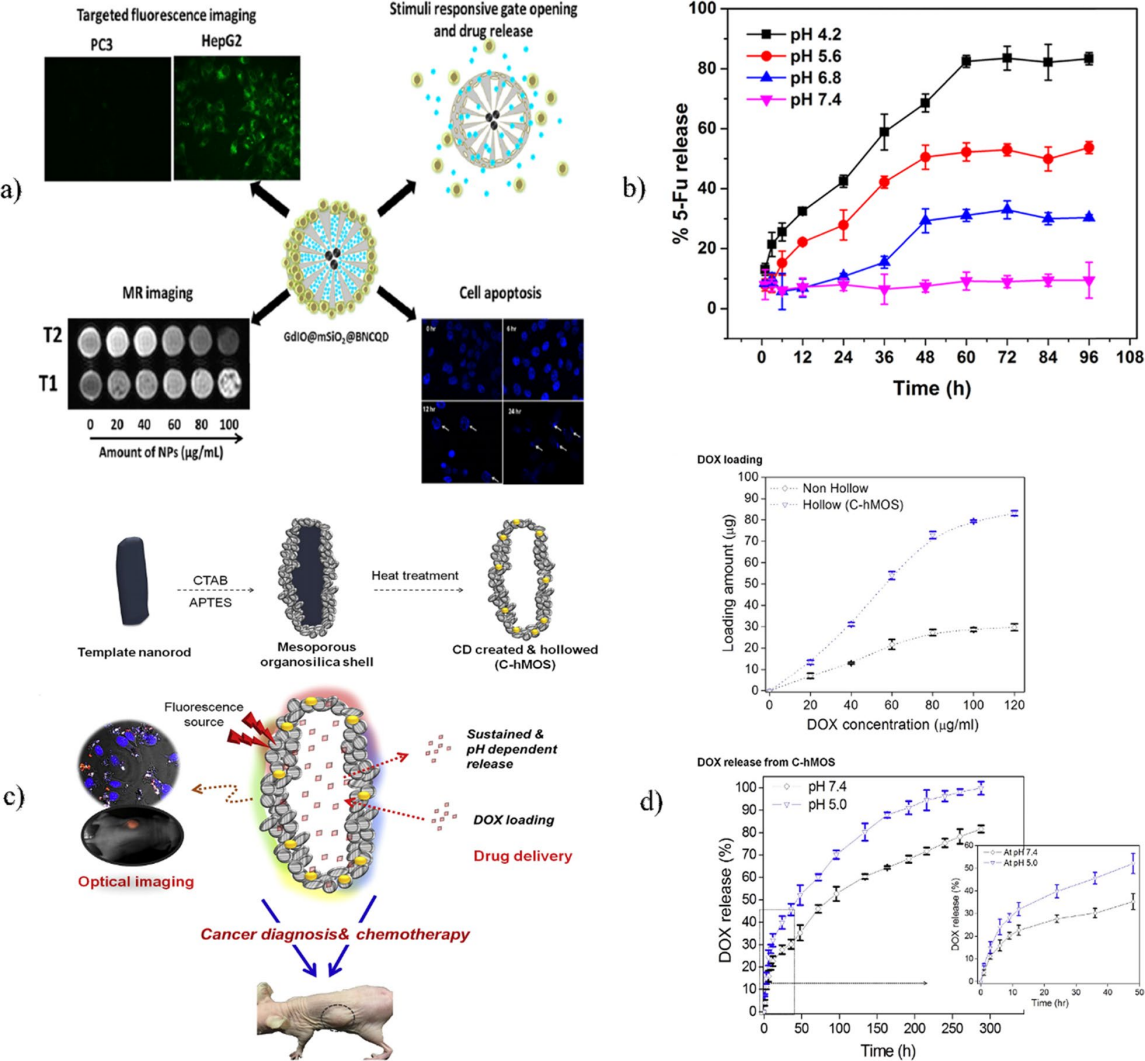
Schematic representation of multifunctional nanoplatform construction for theragnostic applications. **a** Hybrid nanoplatform composed of a core of magnetic gadolinium oxide nanoparticles coated with silica and charged with 5-fluorouracil and carbon quantum dots functionalized with boronic acid as a lid. **b** Graph of drug release at different pH values. Adapted with permission from ref. [230] © Copyright 2018 Elsevier. **c** CD/hollow nanoparticles used for drug delivery and bioimaging. **d** Graph of drug loading and pH-dependent release [151] © Elsevier 2017

This nanoplatform was able to modulate various engineered functions for cancer cell detection and treatment (see Fig. 4a). In this context, the silica shell adsorbed the chemotherapeutic drug and served to graft the BNSCQDs, which acted as pH-dependent gatekeepers that allowed controlled loading and release of the drug, as well as the generation of multifluorescence emission in the range of 370–500 nm, which played an important role in monitoring the interaction of the nanoplatform with the cells. On the other hand, the magnetic nanoparticles granted outstanding relaxivity values of $r_{1} = 10{\text{ mM}}^{ - 1} {\text{s}}^{ - 1}$ and $r_{2} = { }165{\text{ mM}}^{ - 1} {\text{s}}^{ - 1}$ measured by a clinical MRI scanner. The nanoplatform was successfully used for in vitro HePG2 cell detection through the overexpressed sialyl Lewis^a^ (SL^a^) receptor. Additionally, the nanoplatform was successfully used for the release of the drug 5-fluorouracil. To validate the release mechanism, the nanoplatform was subjected to different pH values, showing an optimal percentage of initial release of 13% in 12 h and a final value of 85% in 60 h at a pH of 4.2 (see Fig. 4b). Notably, this work opened a new potential application of CDs combined with diverse nanomaterials, creating an engineered advanced theragnostic device for nanomedical applications [230]. Kang et al. [151] reported the development of CD hollow mesoporous organosilica nanocarriers (C-hMOSs) [170, 232]. The procedure was achieved through the precipitation reaction and surfactant-templated method in the presence of CTAB. The precipitation reaction produced hydroxyapatite (HA) nanoparticles, which were implemented as a core template to create a hollow structure. To fabricate the hollow mesoporous nanoparticles, first, a nonhollow silica structure was synthesized through a modified base-catalyzed solgel process to coat the core template nanoparticles via polymerization of TEOS and APTES. The fluorescent CDs were generated by heating the nonhollow nanoparticles at 400 °C, which were then converted into a hollow-cored structure by removing the core with an acid etching process. The produced multifluorescent hollow-spaced mesoporous silica rods were applied and studied extensively in biological systems (see Fig. 4c). The theragnostic platform showed potential as a drug nanocarrier, in tissue imaging and as a cell marker. To address these capabilities, the nanocarrier was loaded with the chemotherapeutic drug doxorubicin (DOX) and then tested in vitro and in vivo. Interestingly, the nanocarrier exhibited a surface area value of 1087 m^2^/g and pore volume of 0.843 cm^3^/g, which are favorable for drug loading. To verify the theragnostic properties of the nanocarrier, the HeLa cancer cell line was cultured with different concentrations of the DOX-loaded nanoparticles. The results revealed pH-dependent drug release behavior, with faster release at pH 5.0 than at pH 7.4 (see Fig. 4d). The toxic effects of the nanocarrier to cells were studied based on the expression of caspase 3, which was observed by confocal microscopy. Likewise, the quantification of cells positive for the apoptotic marker was performed through flow cytometry, where values of 66% and 91% were found for free DOX and the nanocarrier loaded with DOX. Moreover, the in vivo performance of the nanocarrier was confirmed in MCF-7 tumor-bearing nude mice that were administered the nanocarrier by intratumoral injection. The nanocarrier showed a fluorescent signal at the tumor location and promoted DNA fragmentation as well as tumor growth suppression [151].

**Fig. 5:**
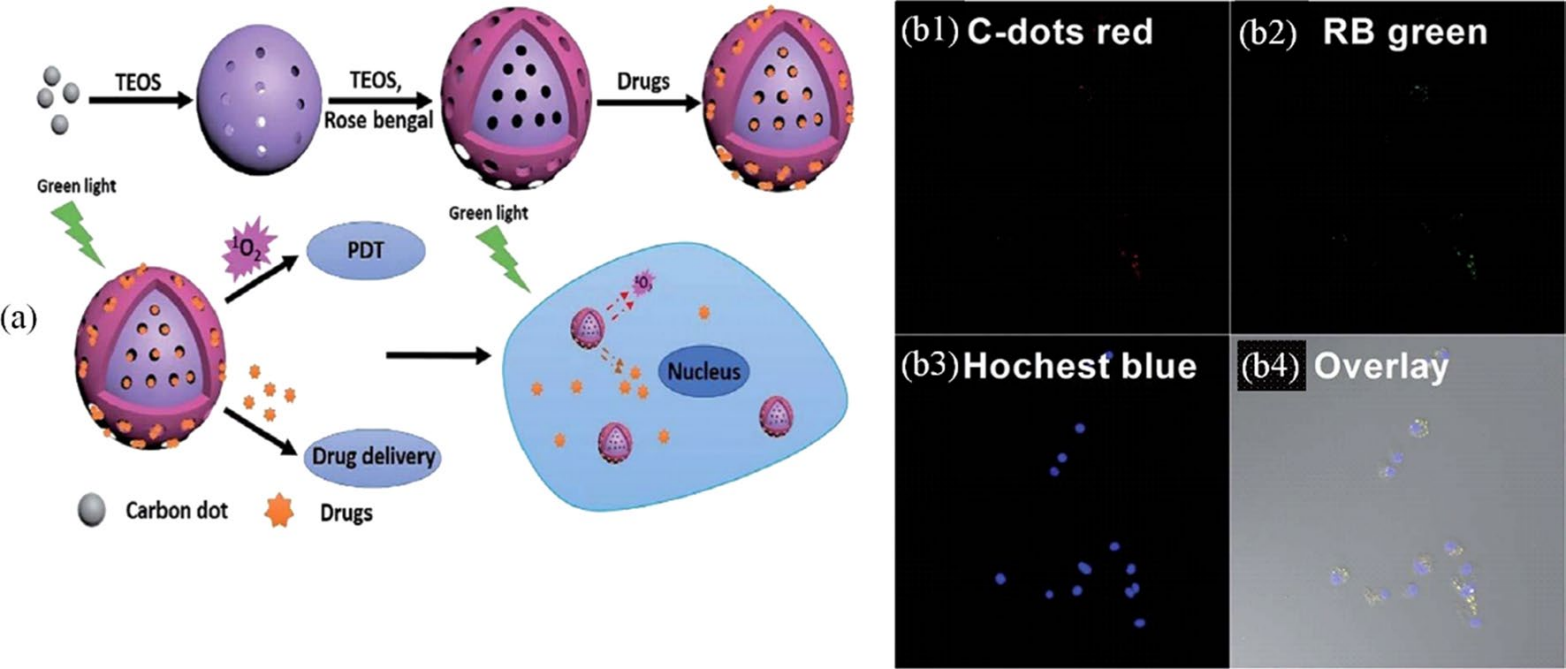
**a** Schematic representation of the multifunctional nanoplatform of silica–CDs. **b** The nanoplatform is localized in the cytoplasm, and the signal is due to the red CDs. All (**b1**–**b4**) images were obtained by confocal microscopy. Reproduced with permission from reference [247]. Copyright 2017 Royal Society of Chemistry

The limitations of the current pharmaceutical formulations for cancer treatment, including antitumor off-target effects, poor solubility, and uncontrolled release, have led to considerable adverse health effects [233, 234]. Thus, there exists an urgent need to improve the efficacy of pharmaceutical treatments because of the high rate of deaths per year associated with cancer [235]. The unique characteristics of materials at the nanoscale facilitate their interaction with biological systems, which allows the above-mentioned issues to be addressed [236–238]. Two of the most exploited biological applications of nanomaterials are bioimaging and drug delivery for cancer therapy [239–246]. Due to their nature, CD–silica nanoparticles can be merged as multimodal nanoplatforms. For example, Liu et al. [247] constructed a versatile nanoplatform that combines some of the properties of different materials. The nanoplatform exploited the photosensitizing properties of Rose Bengal (RB), the high photostability of CDs, and the features of mesoporous silica in a system identified as MSN@C-dots/RB. RB acted as a photosensitizer for photodynamic therapy, CDs acted as a bioimaging agent, and silica served as a carrier for the loading and release of the chemotherapeutic DOX. The nanomaterial was tested against the human lung cancer cell line NCI-H1299 and as a bactericidal agent against *Escherichia coli* (see Fig. 5a). The authors fabricated CDs through a solvothermal method by using dimethylformamide (DMF), urea, and citric acid. Then, the CDs were embedded into mesoporous silica nanoparticles using an oil–water biphasic stratification method, which employed the cationic surfactant cetyltrimethylammonium chloride (CTAC) as a template, TEOS as a silica source, TEA as a catalyst, and 1-octadecene or cyclohexane as the organic solvent. Similarly, the MSN@C-dots/RB nanoparticles were obtained by the same approach adding the photosensitizer RB into the reaction system. During the synthesis, it was observed that at higher doping concentrations of RB or CDs, the morphologies of the nanoparticles were affected; thus, to avoid this drawback, a mesoporous silica shell was epitaxially grown onto the CDs, which produced wormlike morphologies with mesoporous channels that extended to the surface of the nanospheres; these channels increased the loading capacity of DOX and RB. The potential of the nanoplatform for drug delivery and bioimaging was evaluated. First, the increased pore volume, surface area, and mesoporosity caused by epitaxial growth permitted efficient loading of the therapeutic drug and the antibiotic ampicillin into the nanoplatform, which was used for treating the human lung cancer cell lines NCI-H1299 and *E. coli*. The loading and release of DOX were confirmed through pH changes. The percentage of loading was 34.4% (w/w), while the release was pH dependent. A higher percentage of release was reached at pH 5.0, with a percentage value of 50.5%, whereas, for ampicillin, the value was 29.3%. Regarding the optical properties, the MSN@C-dots/RB nanoparticles exhibited excitation-independent photoluminescence and broad emission from 570 to 660 nm. This broad emission was favorable for bioimaging applications. Another highlighted ability of MSN@C-dots/RB was its multifunctionality in chemo/photodynamic therapy. Cells were incubated with the nanoparticles and irradiated at 530 nm and 300 mW cm^−2^ to generate singlet oxygen, ^1^O_2_, which reduced the viability of cancer cells in combination with DOX. Moreover, the nanoparticles loaded with the antibiotic and irradiated showed bacterial inhibition. Additionally, the MSN@C-dots/RB nanoparticles were visualized within the cytoplasm, suggesting that they can be used for monitoring cancer cells (see Fig. 5b1–b4).

**Fig. 6:**
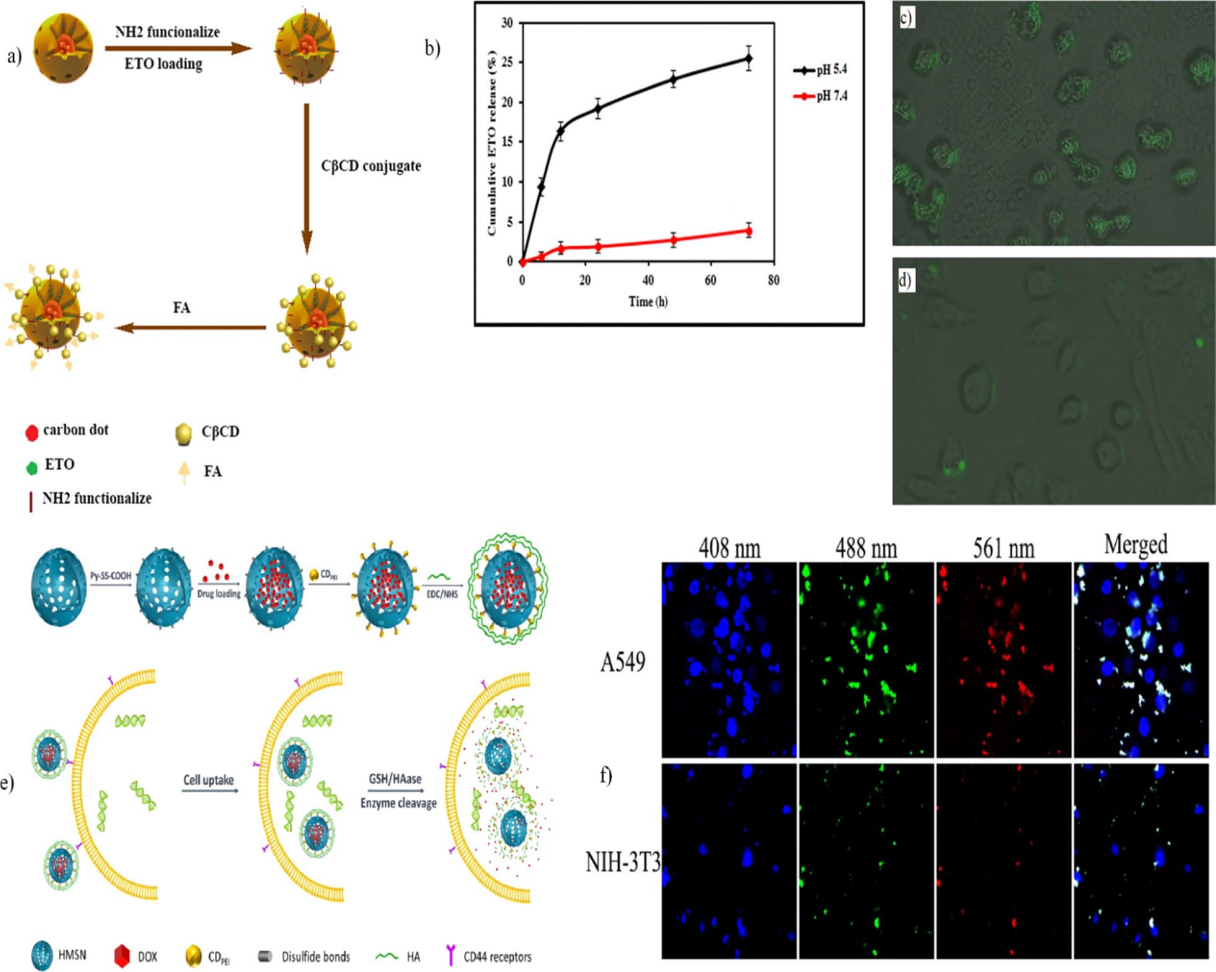
**a** Scheme of the formation of pH-responsive nanocarriers for drug delivery and bioimaging, **b** graph of drug release at different pH values of 5.4 and 7.4, **c**, **d** fluorescence images of endocytosis of FA-C ${\upbeta }$ CD-MSCD-ETO by HeLa and HepG2 cells. Adapted with permission from ref. [252] © 2018 Elsevier. **e** Schematic representation of nanoplatform formation, drug loading, and the mechanism of dual-stimuli drug release. **f** Endocytosis of the nanoplatform by A549 and NIH-3T3 cells. Images obtained by CLSM. Adapted with permission from ref. [225]. Copyright 2017 Elsevier

Currently, diverse research groups have aimed to design nano-DDSs to improve and guarantee a major degree of drug delivery efficiency [132, 133, 248]. For this purpose, researchers can arrange diverse predesigned organic and inorganic functional molecules to form different nanostructures and identify superior results [249, 250]. This type of nanostructure is dominated by supramolecular chemistry interactions including self-assembly, hydrogen bonding, van der Waals forces, dipole–dipole interactions, hydrophobic interactions, and so forth [251]. Therefore, these interactions are a powerful tool for engineering nanomaterials as “gatekeepers,” which can modulate controlled drug delivery. For example, Shirani et al. [252] created a nanocarrier for drug delivery and bioimaging. The nanocarrier traits are conferred by the mesoporous silica and luminescent CDs [252]. In brief, the synthesis was performed by using the pyrolysis route to obtain CDs from glucose and L-aspartic acid. Then, mesosilica-coated CDs (MSCDs) were produced by the solgel process assisted by CTAB and TEOS. Later, the surface of the nanoparticles was modified with APTES to incorporate amine groups. Consequently, to provide multifunctional drug delivery and imaging behavior, the MSCDs were loaded with the antineoplastic agent epipodophyllotoxin (ETO) and conjugated with ${\upbeta }$-cyclodextrin (C ${\upbeta }$ CD) through an N-(3-dimethylaminopropyl)-N-ethylcarbodiimide hydrochloride (EDC)/N-hydroxysuccinimide (NHS) reaction to form an amide linkage. Finally, FA moieties were attached to the conjugated structure (see Fig. 6a). This nanocarrier was then applied to HeLa and Hep2 cells. The results showed that pH-responsive drug release increased in the acidic microenvironment. Furthermore, the folate-decorated nanocarrier interacted with folate receptors (FRs) targeting cancer cells and promoting cellular uptake, which was observed by fluorescence microscopy due to signals emitted by CDs (see Fig. 6b–d).

Zhao et al. [225] developed a DDS that responds to dual endogenous stimuli such as redox and enzyme stimuli. The nanosystem was produced using CDs-PEI and hollow mesoporous silica. The CDs were synthesized through a hydrothermal method by using citric acid and PEI as starting precursors. Then, functionalized hollow mesoporous silica nanoparticles (HMSN-SH) were prepared by an etching process. In particular, ${\text{SiO}}_{2}$ nanospheres were obtained using the Stöber method through TEOS and CTAB and thiol-functionalized by MPTMS. Once the HMSN-SH were obtained, they were further reacted with Py-SS-COOH through a disulfide bond exchange reaction, resulting in modified HMSN-SS-COOH, which was then loaded with DOX and activated with EDC/NHS to covalently graft the positively charged CDs-PEI through an amidation reaction; the final product was obtained after cladding with hyaluronic acid (HA) (Fig. 6e). These HMSN-SS-CD-PEI@HA nanoparticles were used to treat the cancer cell lines A549 and NIH-3T3. The CDs-PEI acted as an imaging agent and played a gatekeeper key role for targeted drug delivery, also conferring controlled drug release. The nanoparticles showed cumulative DOX release at pH values of 7.4 and 5.0 due to the influences of high concentrations of glutathione (GSH) present on the tumor cells, suggesting successful redox-responsive behavior enhanced by HAnase. Considerably, the nanosystem showed potential enhanced theragnostic performance assisted by the outstanding CD fluorescence properties and the verified intrinsic drug cargo loading property of mesoporous silica (Fig. 6f) [225].

## Conclusions

The outstanding physicochemical characteristics of CDs, silica nanoparticles and their derivatives, either as individual particles or combined as nanocomposites, have attracted increasing interest among researchers of diverse disciplines. Exploiting the eminent characteristics of both nanomaterials combined into one nanostructure has been reflected in improvements and enhanced reliability for potential applications in several science fields. The well-documented synthetic routes and the chemical procedures for functionalization of both nanomaterials facilitate the engineering strategy design for combining and fabricating CD–silica nanostructures. In addition, taking advantage of supramolecular interactions as building blocks to produce hybrid nanostructures allows addressing the current drawbacks of DDSs in biological systems, sensing and biosensing. Smart nanohybrid design (mesoporous silica/magnetic nanoparticles/CDs/cancer active drug or mesoporous silica/plasmonic nanoparticles/CDs/cancer active drug) has opened a new way to exploit the characteristic physicochemical properties of nanoscale materials in just one nanodevice. The continual search for new synthetic routes to create nanoarchitectures with novel and unique morphologies, such as 3D dendritic mesoporous nanoparticles, will probably lead to more improvements in recent nanohybrids. New studies and more comprehensive knowledge about the photoluminescence mechanism of CDs will surely arise and will permit better control of their luminescence properties. Interdisciplinary work will lead to new insights and reinforce the promising prospects of CD–silica nanostructures as potential nanoplatforms for the discussed applications.

## Data Availability

Not applicable.

## Abbreviations

AEAPMS: N-(β-Aminoethyl)-γ-aminopropylmethyldimethoxysilane
AIBA: $\alpha ,\alpha^{,}$-Azodiisobutyramidine dihydrochloride
APTES: (3-Aminopropyl)triethoxysilane
APTMS: (3-Aminopropyl)trimethoxysilane
BNSCQD: Boronic acid-modified nitrogen, sulfur codoped carbon quantum dots
CDs: Carbon dots
C-hMOS: CD-induced hollow mesoporous organosilica nanocarriers
CTAB: Cetyltrimethylammonium bromide
CTAC: Cetyltrimethylammonium chloride
DDS: Drug delivery system
DNZ: Diniconazole
DOX: Doxorubicin
EDC: N-(3-Dimethylaminopropyl)-N-ethylcarbodiimide hydrochloride
FRET: Förster resonance energy transfer
GC: Gas chromatography
GSH: Glutathione
HA: Hydroxyapatite
HMSMs: Hollow mesoporous silica microspheres
HMSN-SH: Thiol-functionalized hollow mesoporous silica nanoparticles
HPLC: High-performance liquid chromatography
MIP@CDs: Molecular imprinted polymers—carbon dots
MIPs: Molecularly imprinted polymers
MIT: Molecular imprinting technology
MPTMS: 3-Mercaptopropyltrimethoxysilane
MSCDs: Mesosilica nanoparticle-coated carbon dots
MSMs: Mesoporous silica microspheres
NHS: N-Hydroxysuccinimide
PEI: Polyethyleneimine
PEO: Poly(ethylene oxide)
PPO: Poly(propylene oxide)
PVP: Polyvinylpyrrolidone
Py-SS-COOH: 2-Carboxyethyl-2-pyridyl disulfide
QDs: Quantum dots
SFS: Synchronous fluorescence spectroscopy
SL: Sialyl Lewis
STAB: Stearyltrimethylammonium bromide
TC: Tetracycline
TEA: Triethanolamine
TEOS: Tetraethyl orthosilicate
THMPS: 3-(Trihydroxysilyl)propyl methylphosphonate monosodium salt solution
TMOS: Tetramethyl orthosilicate
TTAB: Tetradecyltrimethylammonium bromide
